# Multimodal Delivery of Isogenic Mesenchymal Stem Cells Yields Synergistic Protection from Retinal Degeneration and Vision Loss

**DOI:** 10.5966/sctm.2016-0181

**Published:** 2016-09-09

**Authors:** Benjamin Bakondi, Sergey Girman, Bin Lu, Shaomei Wang

**Affiliations:** ^1^ Board of Governors Regenerative Medicine Institute, Department of Biomedical Sciences, Cedars‐Sinai Medical Center, Los Angeles, California, USA

**Keywords:** Mesenchymal stem cell transplantation, Retinal pigment epithelium, Stromal derived factor‐1α, Trophic factor receptors, Immunosuppression, Age‐related macular degeneration, Retinitis pigmentosa, Translational research

## Abstract

We previously demonstrated that subretinal injection (SRI) of isogenic mesenchymal stem cells (MSCs) reduced the severity of retinal degeneration in Royal College of Surgeons rats in a focal manner. In contrast, intravenous MSC infusion (MSC^IV^) produced panoptic retinal rescue. By combining these treatments, we now show that MSC^IV^ supplementation potentiates the MSC^SRI^‐mediated rescue of photoreceptors and visual function. Electrophysiological recording from superior colliculi revealed 3.9‐fold lower luminance threshold responses (LTRs) and 22% larger functional rescue area from combined treatment compared with MSC^SRI^ alone. MSC^IV^ supplementation of sham (saline) injection also improved LTRs 3.4‐fold and enlarged rescue areas by 27% compared with saline alone. We confirmed the involvement of MSC chemotaxis for vision rescue by modulating C‐X‐C chemokine receptor 4 activity before MSC^IV^ but without increased retinal homing. Rather, circulating platelets and lymphocytes were reduced 3 and 7 days after MSC^IV^, respectively. We demonstrated MSC^SRI^‐mediated paracrine support of vision rescue by SRI of concentrated MSC‐conditioned medium and assessed function by electroretinography and optokinetic response. MSC‐secreted peptides increased retinal pigment epithelium (RPE) metabolic activity and clearance of photoreceptor outer segments ex vivo, which was partially abrogated by antibody blockade of trophic factors in concentrated MSC‐conditioned medium, or their cognate receptors on RPE. These data support multimodal mechanisms for MSC‐mediated retinal protection that differ by administration route and synergize when combined. Thus, using MSC^IV^ as adjuvant therapy might improve cell therapies for retinal dystrophy and warrants further translational evaluation. Stem Cells Translational Medicine *2017;6:444–457*

**Significance Statement**

Despite hundreds of clinical trials, just one stem cell treatment has been approved for the U.S. market. Additional treatments nearing clinical acceptance use bone marrow mesenchymal stem cells for inflammatory and immune‐related conditions. This is because safety has been established over decades of testing, and cell transplants prolong life‐saving organ and tissue grafts. In the present study, the intravenous delivery of mesenchymal stem cells enhanced the vision rescue from primary cell grafts into diseased retinae. This combined transplant strategy could improve functional outcomes for cell‐based therapies, expand their utility, and expedite their clinical acceptance.

## Introduction

Retinal degenerative diseases, such as age‐related macular degeneration (AMD) and retinitis pigmentosa (RP) affect more than 10 million people in the U.S.; however, current treatment options provide only modest or transient benefit 1, 2. Among the hundreds of clinical trials that have begun to develop stem cell‐based products for treating a multitude of indications, the Food and Drug Administration has granted market authorization to just one product, which contains hematopoietic stem cells (HSCs) 3 that have been used in transplants since 1968 4. The therapies approved abroad that use MSCs for inflammatory and immune‐related conditions such as graft‐versus‐host disease 5 and osteoarthritis are among those nearing U.S. approval 6. Thus, the immunosuppressive properties of MSCs have provided the greatest traction for clinical adoption. Evidence to support immune modulation comes from animal studies that showed intravenous MSC infusion (MSC^IV^) promotes the tolerance of transplanted tissues, thereby prolonging primary graft survival and the duration of functional benefit 7, 8, 9, 10, 11. It is therefore of translational interest to determine whether MSC^IV^ supplementation can likewise enhance the therapeutic benefit of primary stem cell grafts to treat retinal dystrophies.

For RP patients with recessive mutations in the MER proto‐oncogene tyrosine kinase (*MERTK*) gene 12, Royal College of Surgeons (RCS) rats bearing an orthologous *MerTK* mutation 13 represent the ideal model in which to evaluate therapeutic interventions. The retinal pigment epithelium (RPE) is defective in the phagocytosis of diurnally shed photoreceptor outer segments (POSs), which accumulate as toxic debris in the subretinal space. Consequently, photoreceptors are progressively lost, with commensurate vision decline from the third postnatal week to 3 months of age 14, 15. We, and others, have demonstrated protection of RCS retinae by subretinal injection of MSCs (MSC^SRI^) derived from Wharton's jelly 16, umbilical cord tissue 17, or human bone marrow 18. Consistent with the syngeneic graft tolerance proposed for inbred RCS rats without immunosuppression 19, we demonstrated retinal protection through the noninvasive delivery of isogenic MSC^IV^ in immunocompetent RCS rats 20.

Mechanical injury to the retina occurs from hypodermic needle insertion alone during sham injection, which produces transient and focal rescue by local trophic factor expression 21, 22. This sham effect was shown to mobilize and recruit endogenous progenitor cells to the retina; however, the lack of enduring vision rescue indicated that the autonomous response was insufficient 23, 24. Enhanced endogenous progenitor cell integration via cytokine infusion augmented retinal rescue 25, but vision improvement was not demonstrated. Similarly, vision rescue has not been correlated with retinal homing of exogenous cells 26. The pivotal motogen that recruits C‐X‐C chemokine receptor 4 (CXCR4)‐bearing cells to disease or injury sites is C‐X‐C chemokine ligand 12 (CXCL12). CXCL12 conditioning of cultured MSCs enhanced retinal homing, integration, and rescue from acute injury 26. However, cell replacement as a mechanism of retinal protection has been confounded by observations of low MSC engraftment 27. Despite evidence of human MSC differentiation into neurons 28, photoreceptors 29, and RPE 30 after subretinal transplantation in RCS rats, we previously observed only a brief persistence of MSC^SRI^ at injection sites without integration 18. In contrast, we observed long‐term retinal persistence of human neural progenitor cells (NPCs) 31 and induced pluripotent stem cell‐derived NPCs (iNPCs) 32, which compensated for defective RPE by adopting a phagocytic role and preventing POS accumulation. However, we also observed no retinal integration.

In the present study, we addressed whether isogenic MSC^IV^ supplementation could enhance the vision rescue conferred by MSC^SRI^ in the absence of immune suppression. We posited that MSC transplantation by two different routes of administration would control for discrepancies in graft immunogenicity and highlight differences in donor cell function. To investigate whether vision rescue relies on MSC^IV^ homing, we mechanically injured one eye by subretinal injection of balanced salt solution (BSS^SRI^) to induce the sham effect and create a chemotactic source in which competitive migration and function could be compared within each animal. We found that MSC^IV^ potentiated the retinal protection conferred by MSC^SRI^ or BSS^SRI^ through nonredundant mechanisms. Translational implications from combined MSC therapy suggest that MSC^IV^ might serve as a universal adjuvant to improve clinical outcomes for cell‐based therapies.

## Materials and Methods

### Animal Procedures

The institutional animal care and use committee of Cedars‐Sinai Medical Center's comparative medicine department approved animal procedures, which were performed in compliance with the Association for Research in Vision and Ophthalmology Statement for the Use of Animals in Ophthalmic and Vision Research. Dystrophic, pigmented RCS rats received isogenic MSC^IV^ or MSC^SRI^, or both, at degeneration onset (postnatal day [P]21–28). Unilateral MSC^SRI^ (5 × 10^4^ cells) in 2 μl of BSS (Alcon, Fort Worth, TX, http://www.alcon.com) was performed through a scleral incision using a fire‐polished glass pipette (internal bore diameter, 75‐150 μm), attached by rubber tubing to a 25‐μl syringe (Hamilton, Reno, NV, http://www.hamilton.com). Sham injection consisted of 2 μl of BSS^SRI^. At 24 hours after SRI, the rats received MSC^IV^ via the tail vein with 1.25–1.8 × 10^6^ RCS rat MSCs in 500 µl of BSS.

### Visual Function Assessment

Optokinetic response (OKR), electroretinography (ERG), and luminance threshold recording (LTR) were performed according to our published reports 20, 32, 33. OKR permitted noninvasive gross measures of visual acuity as a function of reflexive image stabilization. Scotopic ERGs were used to correlate visually evoked electrical activity with physiological rescue. ERGs were recorded using binocular contact lens electrodes with the Espion E2 System (Diagnosys LLC, Lowell, MA, http://diagnosysllc.com). To measure LTR, we recorded the responses of multiunit neuronal activity in superior colliculi (SC) to light flashes of a 3° area placed in the receptive field while varying the spot brightness. The LTR was defined as the minimal spot luminance capable of eliciting reliable responses with twofold amplitude above background activity. Measurements were performed at 14–18 locations (0.3–0.5‐mm apart) along each side of SC for maximal coverage of the visual field represented on the SC. Data were graphically expressed as the percentage of SC sites at which the LTR was lower or equal to the luminance values indicated by the *x*‐axis and as raw values recorded from individual animals with colored heat map overlay.

### Histology and Immunostaining

Photoreceptor rescue was determined at P60 and P90. The eyes were removed, fixed for 1 hour in 4% paraformaldehyde, subjected to sucrose‐water exchange, embedded in Tissue‐Tek O.C.T. compound (Sakura Finetek Inc., Tokyo, Japan, http://www.sakura‐finetek.com), sectioned to 10 µm via a cryostat and stained with 0.4% cresyl violet acetate to examine retinal lamination. CXCR4 immunolabeling was performed with antibody clone 12G5 at 1:100 dilution (Abcam Inc., Cambridge, U.K., http://www.abcam.com).

### MSC Isolation and Culture

MSCs were derived from 6–8‐week‐old RCS rats, as previously described 20. For proliferation assays, MSCs were quantified by fluorescent labeling of nucleic acids (CyQuant GR fluorescent dye; Thermo Fisher Scientific Life Sciences, Waltham, MA, http://www.thermofisher.com) in multiwell plates (Envision Multilabel plate reader; Perkin Elmer, Waltham, MA, http://www.perkinelmer.com). MSC‐conditioned medium (CdM) was produced, as previously described 34, concentrated using Amicon Ultra Columns of 3‐kDa pore size (EMD Millipore, Bedford, MA, http://www.emdmillipore.com), and frozen at −80°C until use. Multilineage MSC differentiation after CXCR4 modulation was performed, as previously described 34.

### MSC Migration Analysis Ex Vivo and In Vivo

CXCR4 was stimulated in MSCs at passages 4 to 5 with 50 ng/ml recombinant rat CXCL12 (PeproTech, Rocky Hill, NJ, http://www.peprotech.com) or inhibited with 10 μM 1,1′‐[1,4‐phenylenebis(methylene)]bis‐1,4,8,11‐tetraazacyclotetradecane (AMD3100; Sigma‐Aldrich, St. Louis, MO, http://www.sigmaaldrich.com) or exposed to BSS (control) for 2 hours. MSCs were washed (2× phosphate‐buffered saline [PBS]), cultured for an additional 4 hours, and then enzymatically detached from cultureware (0.05% trypsin EDTA; Corning Inc., Corning, NY, http://www.corning.com), manually quantified, resuspended in BSS, and used for MSC^IV^ or plated (5 × 10^4^) onto Millicell inserts of 12‐µm pore size (EMD Millipore) in triplicate for migration assays. Replicate aliquots from each treatment were fixed or permeabilized (4% paraformaldehyde/Triton X‐100), stained for immunofluorescence and flow cytometry intracellular staining (FACSAria III, BD Biosciences, San Jose, CA, http://www.bdbiosciences.com). MSCs migrated overnight toward 50 ng/ml CXCL12 in basal medium (BM) containing Dulbecco's modified Eagle's medium (DMEM) with 100 U/ml penicillin/streptomycin (Thermo Fisher). in the bottom chamber. Transwell membranes were washed with PBS, nonmigrated cells were removed via a cotton‐tipped applicator, fixed/permeabilized (10% formalin/Triton X‐100), and the membranes were removed and coverslipped on glass slides (VWR International, LLC, Randor, PA, http://www.vwr.com) using mounting media containing nucleic acid fluorescent dye 4′,6‐diamidino‐2‐phenylindole (DAPI) to identify migrated cells (Vector Laboratories, Burlingame, CA, http://www.vectorlabs.com). Migration was quantified by averaging the cell number from 10 random fields of view at ×40 magnification using Java‐based imaging software (ImageJ, version 1.46; National Institutes of Health, Bethesda, MD, http://www.imagej.nih.gov). BM served as the negative control, and BM with 30% fetal bovine serum (FBS; Atlanta Biologicals Inc., Flowery Branch, GA, http://www.atlantabio.com) served as the positive control.

Fluorescent cell membrane dyes PKH26 and PKH67 (Sigma‐Aldrich) were used to label MSCs immediately before injection. Retinal imaging was performed on flat‐mount dissections or 10‐µm cryosections. For flow cytometry, the eyes were removed and stored at 4°C in PBS (pH 7.4) and dissociated into single‐cell suspension by 20 minutes of incubation at 37°C with enzymatic solution consisting of Ca^2+^/Mg^2+^‐free PBS, 20 U/ml papain, and 0.5 mM l‐cysteine (Worthington Biochemical Corp., Lakewood, NJ, http://www.worthington‐biochem.com). Fluorescent cells were detected via LSR Fortessa Analyzer (BD Biosciences). To determine whether MSC‐CdM rescued retinal function, the rats received unilateral SRI of serum‐free 35× CdM (CdM^SRI^) collected from passage 3 to 5 RCS‐MSCs. A complete blood count (CBC) was performed on days 1, 3, 7, and 14 on peripheral blood extracted via the tail vein using the Hemavet 950FS (Drew Scientific, Miami Lakes, FL, http://www.drew‐scientific.com). Tissues were evaluated for migration of PKH^+^ MSCs on day 1, 3, or 7 in retinal cryosections.

### RPE Assays

RPE cultures were established from P10 RCS rat retinae, seeded at 1,000 per well in 96‐well clear‐bottom tissue culture fluorescence plates (Corning Inc.), and expanded in growth medium (GM) containing Dulbecco's modified Eagle's medium, 10% fetal bovine serum, 100 U/ml penicillin/streptomycin for 2 weeks before bioassays. RPE metabolic activity was measured by cellular dehydrogenase enzyme conversion of 3‐(4,5‐dimethylthiazol‐2‐yl)‐5‐(3‐carboxymethoxyphenyl)‐2‐(4‐sulfophenyl)‐2H‐tetrazolium substrate to formazan by colorimetric assay (CellTiter 96 AQueous One; Promega Corp., Madison, WI, http://www.promega.com).

To determine RPE phagocytosis after CdM exposure, bovine POSs (InVision BioResources, Seattle, WA, http://www.invisionbio.com) were labeled with fluorescein‐5‐isothiocyanate isomer‐I (FITC; Thermo Fisher) and fed to RPE. RPE were washed (2× PBS), cultured overnight in BM, exposed to 5× MSC‐CdM for 24 hours, and incubated for 18 hours following FITC‐POS addition at 1.44 μg per well. Total fluorescence was measured after three PBS washes to remove unbound POSs (Envision Multilabel plate reader; Perkin Elmer). Internalized POSs were quantified by ImageJ colocalization function after fixation/permeabilization (4% paraformaldehyde/Triton X‐100, 20 minutes) via lysosomal maker immunolabeling for cathepsin‐D 1:250 (Santa Cruz Biotechnology Inc., Dallas, TX, http://www.scbt.com), and normalized by cell number (DAPI). RPE unexposed to FITC‐POS served as baseline fluorescence control. Samples were measured in triplicate or quadruplicate.

For trophic factor and receptor blocking/neutralization studies, the following antibodies were used at 5 µg/ml: mouse immunoglobulin (Ig) G_1_ isotype control, brain‐derived neurotrophic factor (BDNF) neutralizing (Thermo Fisher), fibroblast growth factor (FGF)‐2, vascular endothelial growth factor (VEGF)‐A, CXCL12, glial‐derived neurotrophic factor (GDNF), BDNF, ciliary neurotrophic factor (CNTF) p75 nerve growth factor receptor (Santa Cruz Biotechnology Inc., Dallas, TX, http://www.scbt.com), connective tissue growth factor (CTGF; Bioss Antibodies Inc., Woburn, MA, http://www.biossusa.com), nerve growth factor (NGF)/proNGF, tropomyosin receptor kinase (Trk)A, TrkB, TrkC, glial cell‐derived neurotrophic factor receptor α1, rat p75NTR (Alomone Labs, Inc., Jerusalem, Israel, http://www.alomone.com). Recombinant peptides CXCL12, basic FGF (bFGF; PeproTech), and BDNF (Sigma‐Aldrich) were used at 100 ng/ml in BM containing 0.1% NaN_3_ for ex vivo assays.

### Statistical Analysis

Student's *t* tests were performed using two‐tailed distribution and two‐sample unequal variance (heteroscedastic) to compare LTR values averaged for each eye and raw OKR values from individual eyes. To determine the relative treatment efficacy, values from treated and contralateral (untreated) eyes were compared in individual animals and are expressed as the percentage of improvement, with the animals grouped by treatment. Error bars in animal experiments represent SEM and in ex vivo assays, SD. Statistical significance has been indicated according to convention: ∗, *p* ≤ .05; ∗∗, *p* ≤ .01; ∗∗∗, *p* ≤ .001; ^†^, *p* ≤ .05; ^††^, *p* ≤ .01; ^†††^, *p* ≤ .001.

## Results

### Synergistic Efficacy and Morphological Rescue From Combined MSC Transplantation

**Figure 1:**
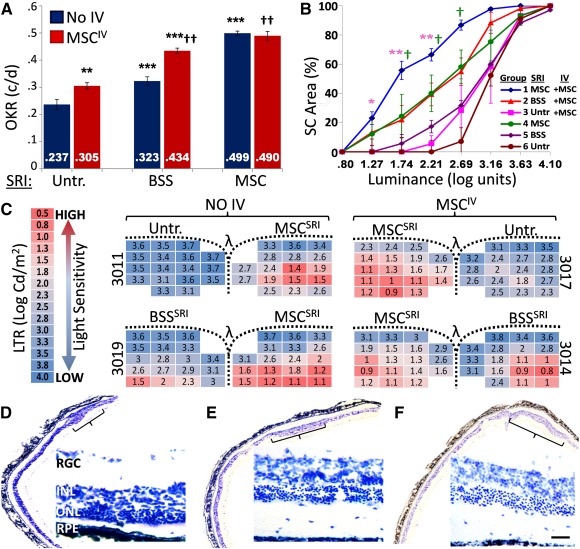
Synergistic efficacy from combined MSC treatment. Postnatal day (P)21–P25 Royal College of Surgeons rats received singular treatment (BSS^SRI^, MSC^SRI^, or Untr.; blue bars) or treatment combined with MSC^IV^ (red bars) and assessed at P90. **(A):** Visual acuity from MSC^SRI^ was 54% higher than with BSS^SRI^ and 110% higher than with Untr. controls. BSS^SRI^ resulted in 36% higher visual acuity compared with Untr. eyes (blue bars). MSC^IV^ supplementation enhanced the visual acuity of Untr. eyes by 29%, BSS^SRI^‐treated eyes by 34%, but not MSC^SRI^‐treated eyes. **(B):** Greater retinal sensitivity to light stimulation (lower luminance threshold) was observed for all subretinal treatments when supplemented with MSC^IV^. Combined MSC^SRI^ and MSC^IV^ treatment (blue line) produced the highest number of responding foci compared with singular treatments MSC^SRI^ (green line) or MSC^IV^ (orange line) at the indicated luminance thresholds. **(C):** Representative LTR from individual animals show retinotopic light sensitivity by heat map indicating focal hot spots (red) as less than 33% of the mean value. The functional rescue area from MSC^SRI^ increased 22% with MSC^IV^ supplementation, corresponding to 3.9‐fold increased total retinal sensitivity. Similarly, MSC^IV^ increased the rescue area from BSS^SRI^ by 27% and retinal sensitivity 3.4‐fold. **(D–F):** Phase‐contrast microscopy images of cresyl violet stained retinae show distinct focal versus panoptic photoreceptor protection that correlates with OKR and LTR data. **(D):** Rats that received MSC^SRI^ alone had five to seven rows of photoreceptor nuclei exclusively at the injection site. **(F):** In contrast, BSS^SRI^ with MSC^IV^ rescued approximately six rows of photoreceptors over a larger area. **(E):** MSC^SRI^ with MSC^IV^ produced greater and more widespread photoreceptor preservation compared with either MSC treatment alone. Brackets indicate focal rescue area. Scale bar = 100 µm. Asterisks correspond to statistically significant differences within MSC^IV^ and MSC^SRI^ groups; daggers correspond to differences between groups. Abbreviations: BSS, balanced salt solution; c/d, cycles per degree; Cd, cycles per degree; INL, inner nuclear layer; IV, intravenous; LTR, luminance threshold response; MSC, mesenchymal stem cell; OKR, optokinetic response; ONL, outer nuclear layer; PR, photoreceptor layer; RGC, retinal ganglion cells; RPE, retinal pigment epithelium; SC, superior colliculi; SRI, subretinal injection; Untr., untreated.

We determined whether MSC^IV^ administration increases the vision rescue conferred by MSC^SRI^ transplantation. We first compared subretinal treatments alone, in which rats received MSC^SRI^ (*n* = 5), BSS^SRI^ (vehicle control, *n* = 6), or no treatment (*n* = 6). As singular treatments, visual acuity was higher from MSC^SRI^ than either BSS^SRI^ (*p* < .001) or untreated controls (*p* < .001; Fig. 1A, blue bars). The higher visual acuity in BSS^SRI^ compared with the untreated eyes illustrated the autonomous protection from the sham effect (*p* < .01).

Using the combined treatment strategy, MSC^IV^ with MSC^SRI^ (*n* = 6) produced the highest visual acuity (vs. BSS^SRI^, *p* < .01; vs. untreated, *p* < .01; Fig. 1A, red bars). The acuity from MSC^SRI^ alone was similar to that of wild‐type rats and did not increase further after MSC^IV^. MSC^IV^ combined with BSS^SRI^ increased the efficacy of the BSS^SRI^‐mediated sham effect by 34% (*p* < .01). This represented the greatest functional improvement from MSC^IV^ supplementation.

Detailed analysis of visual function rescue by LTR indicated synergistic improvement from MSC^IV^ supplementation (Fig. 1B **)**. Bilateral electrophysiological recordings from SC represented approximately 80% of the animal's visual field. The cumulative LTR distribution curve is logarithmic sigmoidal in shape and becomes horizontally exaggerated toward the *y*‐axis as light sensitivity increases. Untreated RCS rats at P90 showed LTR values of 2.7 log units or higher above background (0.02 log unit). In contrast, congenic wild‐type rats typically show ≤0.5 log unit 35. Combined MSC^SRI^ with MSC^IV^ treatment significantly increased retinal sensitivity to light (lower LTR value) compared with MSC^SRI^ or MSC^IV^ singular treatments at the threshold values indicated. LTR did not differ between MSC^SRI^ alone versus BSS^SRI^ combined with MSC^IV^.

A heat map overlay of LTR values represent increasing light sensitivity from low (blue) to high (red), with hot spots of focal rescue defined as values less than 33% of the mean from both colliculi of each animal (Fig. 1C). The hot spot area in MSC^SRI^‐treated eyes increased from 17 of 83 positions (20%) to 36 of 84 positions (43%) by MSC^IV^ supplementation (*p* < .05). The larger rescue area corresponded to a 3.9‐fold increase in total retinal sensitivity (1.85 ± 0.11 vs. 2.44 ± 0.20 log units; *p* < .05). MSC^IV^ supplementation of BSS^SRI^ increased the hot spot area from 8 of 50 (16%) to 21 of 47 positions (44%; *p* < .05) and increased sensitivity 3.4‐fold (2.37 ± 0.17 vs. 2.91 ± 0.02 log units; *p* < .05). LTR consistently showed an increased size of rescue areas from MSC^IV^ supplementation (supplemental online Fig. 1). Photoreceptor rescue areas from MSC^SRI^ alone (Fig. 1D) were larger when combined with MSC^IV^ (Fig. 1E) and when BSS^SRI^ was combined with MSC^IV^ (Fig. 1F).

### Enhanced MSC^IV^ Homing Potentiated Photoreceptor and Vision Rescue

**Figure 2:**
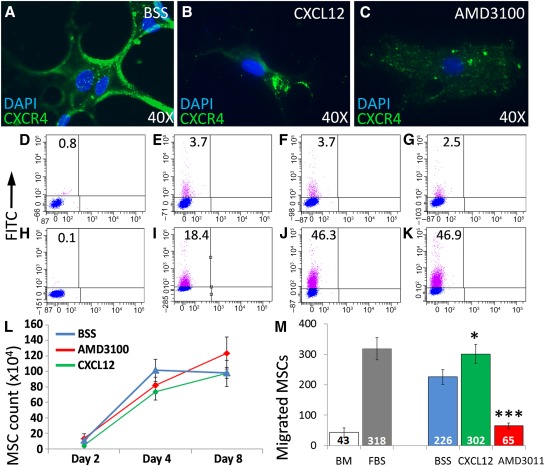
CXCR4 activity modulation alters MSC chemotaxis ex vivo without adverse effects. To confirm the ability of cultured MSCs to respond to homing signals, we reduced MSC chemotaxis in vitro by CXCR4 antagonism and increased it by CXCR4 stimulation. **(A–C):** Fluorescent immunolabeling of MSCs confirmed similar CXCR4 expression after exposure to BSS **(A;** control), CXCL12 **(B)**, or AMD3100 **(C)**. **(D–K):** Flow cytometry dot plots of replicate MSC cultures showed low CXCR4 surface expression after BSS (control) treatment **(E)** that was not different from CXCL12 **(F)** but was slightly reduced with AMD3100 **(G)**. Cell permeabilization before immunostaining showed that compared with BSS **(I)**, intracellular CXCR4 increased with exposure to CXCL12 **(J)** or AMD3100 **(K)**. **(D, H):** Unstained controls. CXCR4 activity modulation did not provide a cell‐dose advantage, as MSC proliferation did not differ between groups over 8 days after treatment **(L)**. MSC migration through Transwell membranes toward CXCL12 was evaluated by DAPI^+^ nuclei quantification in 10 random fields of view at ×40 magnification Two‐way analysis of variance. **(M):** Graphic representation showing that CXCL12 simulation increased MSC migration compared with BSS, and AMD3100 treatment reduced it. ∗, *p* < .05; ∗∗∗, *p* < .001; Student's *t* test. Abbreviations: AMD3100, 1,1′‐[1,4‐phenylenebis(methylene)]bis‐1,4,8,11‐tetraazacyclotetradecane; BM, basal medium (negative control); BSS, balanced salt solution; CXCR4, C‐X‐C chemokine receptor 4; DAPI, 4′,6‐diamidino‐2‐phenylindole; FBS, BM containing 30% fetal bovine serum (positive control); FITC, fluorescein‐5‐isothiocyanate isomer‐I; MSC, mesenchymal stem cell.

**Figure 3:**
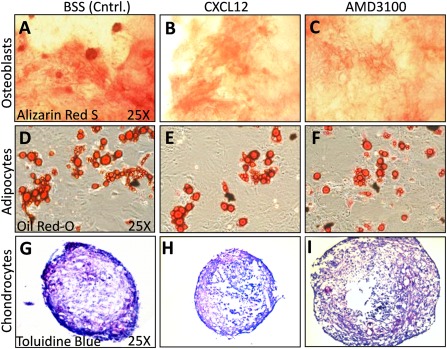
Royal College of Surgeons rat mesenchymal stem cells (MSCs) retain multipotency after C‐X‐C chemokine receptor 4 modulation. To control for changes in MSC multipotency from ex vivo treatments, we compared the differentiation potential of the MSCs used for intravenous MSCs to form osteoblasts, adipocytes, and chondrocytes. **(A–F):** Phase‐contrast microscopy images of confluent MSC cultures exposed to differentiation media for 3 weeks. Histological detection of calcium deposition from osteoblasts **(A–C;** alizarin red S) and lipid formation by adipocytes **(D–F;** Oil Red O) shown. Collagen formation by chondrocytes shown in 10‐µm sections from micromass pellets **(G–L;** toluidine blue sodium borate). MSCs exposed to CXCL12 **(A, D, G)**, AMD3100 **(B, E, H)**, or BSS (Cntrl.; **C, F, I)** showed similar differentiation capacity. Abbreviations: AMD3100, 1,1′‐[1,4‐phenylenebis(methylene)]bis‐1,4,8,11‐tetraazacyclotetradecane; BSS, balanced salt solution; Cntrl., control; CXCL12, C‐X‐C chemokine ligand 12.

To determine whether retinal recruitment after MSC^IV^ is important for vision rescue, we altered the ability of MSCs to home before MSC^IV^. We exposed MSCs to CXCR4 agonist (CXCL12) or antagonist (AMD3100) and confirmed altered migration toward CXCL12 ex vivo and controlled for additional changes to MSC function that might affect graft potency. We first confirmed that MSCs can respond to CXCL12 by their expression of the CXCR4 via immunocytochemistry (Fig. 2A–2C). Extracellular CXCR4 expression on MSCs was shown to decrease in culture 36, but intracellular stores traffic to the cell surface after cytokine exposure 37 and restore MSC tropism to ischemic tissues via CXCL12 signaling 38. By flow cytometry, extracellular CXCR4 levels did not differ by treatment, but intracellular CXCR4 levels increased with CXCL12 or AMD3100 exposure (Fig. 2D–2K). We excluded the possibility that CXCL12 treatment increased proliferation and provided a cell‐dose advantage by demonstrating no change to MSC growth over 8 days in culture (Fig. 2L). MSC chemotaxis was confirmed to increase with CXCL12 stimulation (*p* < .05) and decrease with AMD3100 treatment (*p* < .001; Fig. 2M). Finally, we evaluated changes in MSC multipotency after treatment and did not detect differences in osteogenic (Fig. 3A–3C), adipogenic (Fig. 3D–3F), or chondrogenic (Fig. 3E–3G) differentiation.

**Figure 4:**
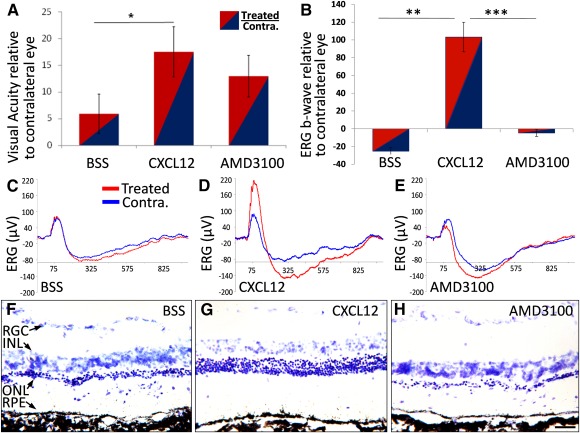
Enhanced intravenous mesenchymal stem cell infusion (MSC^IV^) homing potentiates photoreceptor and vision rescue. Postnatal day (P)21–P25 Royal College of Surgeons rats received unilateral subretinal injection of balanced salt solution, followed by CXCL12‐conditioned MSC^IV^ 24 hours later, with function assessed at P60. **(A):** Raw optokinetic response measurements in units of cycles per degree were normalized to the endogenous control of Contra. (untreated) eyes in individual animals. Rats that received CXCL12‐conditioned MSC^IV^ improved visual acuity by 18% compared with contralateral eyes, which significantly differed from that of BSS‐conditioned MSC^IV^. BSS‐ and AMD3100‐conditioned MSC^IV^ did not significantly improve visual acuity. **(B):** ERG maximal b‐wave amplitudes in treated eyes were normalized to that of Contra. eyes. The b‐wave responses were 103% higher with CXCL12‐conditioned MSC^IV^, but AMD3100‐ and BSS‐conditioned MSC^IV^ did not significantly alter function. **(C–E):** Representative traces from treated eyes (red) and Contra. eyes (blue) from animals that received MSC^IV^ conditioned with BSS **(C)**, CXCL12 **(D)**, or AMD3100 **(E)**. **(F–H):** Photomicrographs of cresyl violet‐stained retinae that received MSC^IV^ illustrate pathological differences. Retinal areas inferior to the injection site showed greater ONL thickness from MSC^IV^ conditioned by CXCL12 **(G)** compared with BSS **(F)** or AMD3100 **(H)**. Scale bar = 100 µm. Abbreviations: AMD3100, 1,1′‐[1,4‐phenylenebis(methylene)]bis‐1,4,8,11‐tetraazacyclotetradecane; BSS, balanced salt solution; Contra., contralateral; CXCL12, C‐X‐C chemokine ligand 12; ERG, electroretinography; INL, inner nuclear layer; ONL, outer nuclear layer; RGC, retinal ganglion cells; RPE, retinal pigment epithelium.

To determine whether MSC chemotaxis contributed to vision rescue, we stimulated or inhibited CXCR4 activity before MSC^IV^ (Fig. 2) and tested the vision at P60. MSCs were exposed to CXCL12 (*n* = 9), AMD3100 (*n* = 6), or BSS (control; *n* = 6; Fig. 4). Because the sham effect increased from MSC^IV^, we performed BSS^SRI^ in one eye to compare the vision improvement between the homing‐modulating treatments. Visual function in BSS^SRI^‐treated eyes was normalized to contralateral (untreated) eyes in each animal. Visual acuity increased 18% from CXCL12‐conditioned MSC^IV^ (*p* < .05; Fig. 4A). AMD3100 conditioning of MSCs did not alter the acuity relative to the contralateral eyes compared with CXCL12 or BSS conditioning (*p* > .05).

Focal retinal rescue is sufficient to elicit a head‐tracking response, but OKR less accurately distinguishes total anatomical/physiological rescue compared with ERG. b‐Wave amplitudes were higher from infusion of CXCL12‐conditioned MSCs compared with BSS (*p* < .01) or AMD3100 conditioning (*p* < .01; Fig. 4B). Representative ERG traces showed lower b‐wave amplitudes in rats that received AMD3100‐ or BSS‐conditioned MSCs compared with CXCL12‐conditioned MSCs (Fig. 4C–4E). Histological assessment of these retinae showed the most photoreceptor rescue with CXCL12‐conditioned MSC^IV^ and the least with AMD3100‐conditioned MSC^IV^ (Fig. 4F–4H).

### Limited Retinal Recruitment of CXCL12‐Stimulated MSC^IV^

**Figure 5:**
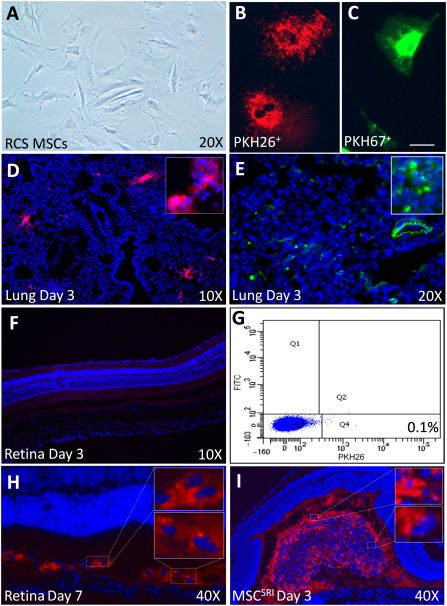
Limited MSC detection in the retina after intravenous infusion of MSCs (MSC^IV^). **(A):** Phase‐contrast microscopy of cultured MSCs at 70% confluence before C‐X‐C chemokine ligand 12, 1,1′‐[1,4‐phenylenebis(methylene)]bis‐1,4,8,11‐tetraazacyclotetradecane, or balanced salt solution (BSS) conditioning. MSCs labeled with PKH26 **(B;** 598‐nm λ‐emission, red/orange) or PKH67 **(C;** 480‐nm λ‐emission, green) showed no cell toxicity, morphological changes, or retained fluorescence over 10 days in vitro. Scale bar = 25 µm. **(D, E):** Lung tissue collected 3 days after MSC^IV^ showed PKH67^+^ MSCs proximal to autofluorescent bronchi‐alveolar microvilli (PKH26 **[D]** and PKH67 **[E]**; insets show magnified views). **(F):** Representative retinal image from rat in **(D)** using identical gain and exposure settings exemplifies typical donor cell absence 3 days after MSC^IV^ (*n* = 6). **(G):** Representative flow cytometry dot plot showing single‐cell dissociated retina quantification with low (0.1%) PKH26^+^ MSC^IV^ detection on day 3. **(H):** Sporadic PKH26^+^ MSCs were observed in the subretinal space on day 7, proximal to the site of subretinal injection of BSS colocalized with 4′,6‐diamidino‐2‐phenylindole‐positive nuclei (insets). **(I):** For comparison, 1 × 10^4^ PKH26 MSC^SRI^ are shown aggregated in the subretinal space on day 3, with similarly eccentric PKH26^+^ puncta (insets, magnified views). Abbreviations: FITC, fluorescein‐5‐isothiocyanate isomer‐I; MSC, mesenchymal stem cell; RCS, Royal College of Surgeons; SRI, subretinal injection.

We investigated whether the enhanced vision from CXCL12‐conditioned MSC^IV^ corresponded with retinal recruitment. We had previously demonstrated MSC recruitment via inner retinal vessels 2 weeks after MSC^IV^ 20 and, in the present study, evaluated homing at 3 and 7 days in BSS^SRI^‐treated eyes. MSCs (Fig. 5A) were labeled with PKH26 or PKH67 and used for MSC^IV^ or culture expanded to monitor fluorescence diminution over 10 days (Fig. 5B, 5C). On day 3, PKH26^+^ MSCs were frequently detected in cryosections of lung tissue (Fig. 5D, 5E) and by flow cytometry of peripheral blood but with low frequency in bone marrow aspirates (supplemental online Fig. 2). In contrast, PKH26^+^ MSCs were absent in retinal sections (Fig. 5F) and rarely detected by flow cytometry of dissociated neural retinae (Fig. 5G). PKH26^+^ MSCs were sporadically detected in the retinal sections by day 7, indicated by DAPI^+^ colocalization with PKH26^+^ fluorescent puncta (Fig. 5H), similar in appearance to the subretinal injected PKH26^+^ MSCs (1 × 10^4^) on day 3 used for illustration (Fig. 5I).

### Evidence for MSC Trophic Support of Visual Function In Vivo and RPE Phagocytosis Ex Vivo

**Figure 6:**
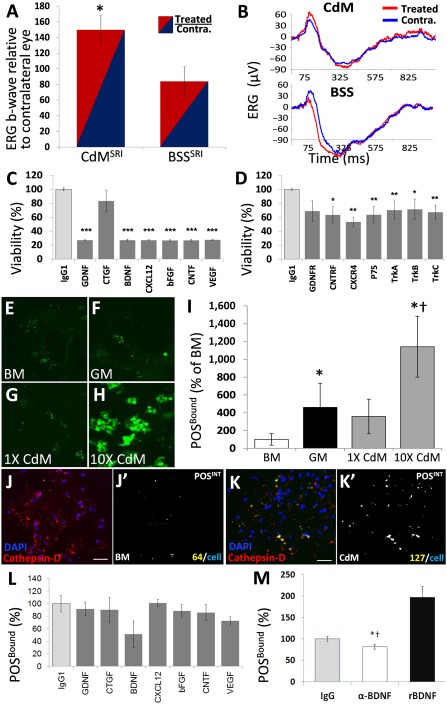
Mesenchymal stem cell (MSC)‐mediated trophic support of visual function in vivo and retinal pigment epithelium (RPE) phagocytosis capacity ex vivo. **(A):** ERG b‐wave amplitudes were higher in eyes treated with 35× MSC‐CdM^SRI^ (normalized to Contra. Eyes; *n* = 3), compared with BSS^SRI^ (*n* = 5) at postnatal day 60 (*p* < .05). **(B):** ERG traces show higher b‐wave amplitude from CdM^SRI^ (66.8 µV) versus BSS^SRI^ (24.6 µV). **(C, D):** Metabolic viability in Royal College of Surgeons RPE increased 24 hours after exposure to 5× CdM, which was abrogated by preincubating CdM with blocking antibodies to select trophic factors **(C)** or by preincubating RPE cultures with antibodies to trophic factor receptors **(D)**. CdM enhanced the total POS^Bound^ **(E–H,** quantified in **I)**. **(J, K):** POS^INT^ colocalization with phagolysosome marker, cathepsin‐D, in BM (**J**, colocalized area in **J’)** was increased by CdM exposure **(K,** colocalized area in **K’)**. **(L):** The peptides in **(C)** were screened for possible contribution to POS^Bound^. **(M):** Total POS^Bound^ was abrogated with BDNF neutralization (82% ± 5% of IgG_1_ control; *, *p* < .05) and was restored with the addition of 50 ng/ml BDNF to BM (197% ± 25% of IgG_1_ control; ^†^, *p* < .05). Abbreviations: BDNF, brain‐derived neurotrophic factor; bFGF, basic fibroblast growth factor; BM, basal medium; BSS, balanced salt solution; CdM, MSC‐conditioned medium; CNTF, ciliary neutrophoric factor; CNTFR, CNTF receptor; Contra., contralateral (untreated); CTGF, connective tissue growth factor; CXCL12, C‐X‐C chemokine ligand 12; CXCR4, C‐X‐C chemokine receptor 4; DAPI, 4′,6‐diamidino‐2‐phenylindole; ERG, electroretinography; GDNF, glial‐derived neurotrophic factor; GDNFR, GDNF receptor; GM, growth medium; IgG, immunoglobulin G; IgG1, immunoglobulin G1; POS^BOUND^, photoreceptor outer segments bound to RPE; POS, photoreceptor outer segments; POS^INT^, RPE‐internalized POS; rBDNF, brain‐derived neurotrophic factor receptor; SRI, subretinal injection; TrkA, TrkB, TrkC, tropomyosin receptor kinase A, B, C; VEGF, vascular endothelial growth factor.

Because few MSCs were detected in retinae from MSC^IV^, we determined whether the presence of MSCs is needed to rescue vision. Low donor cell engraftment opposes cell replacement as a central mechanism of MSC protection 39, 40. The use of MSC‐CdM eliminated the possibility of exogenous cell engraftment as an explanation for therapeutic improvement following injury and, instead, implicated paracrine support by secreted growth factors, cytokines, neurotrophins, and peptide hormones 34, 41. To investigate whether MSC^SRI^ rescued vision through trophic support, we injected 35× concentrated conditioned medium from MSCs (CdM^SRI^) or BSS^SRI^ (control) and assessed vision at P60. ERG b‐wave amplitudes were 50% higher in CdM^SRI^‐treated eyes (relative to untreated contralateral eyes) compared with a 16% decrease with BSS^SRI^ (*p* < .05; Fig. 6A). Representative ERG traces showed higher b‐wave amplitudes in the CdM^SRI^‐treated eyes (red) compared with the contralateral eyes (blue) and compared with BSS^SRI^ (Fig. 6B).

We next investigated whether peptides in CdM affect RPE ex vivo because retinal degeneration in RCS rats is a consequence of phagocytosis dysfunction. We selected trophic factors and their receptors to test by antibody‐blocking assays based on their reported protection of RCS retinae. Pathological rescue was shown with bFGF 42, GDNF 43, BDNF 44, VEGF 45, and CNTF 20. In contrast, CXCL12 rescued photoreceptors in a model of retinal injury 46. Uniquely, CTGF was shown to regulate RPE transdifferentiation 47 and cooperate with growth factor signaling for tissue protection in vivo 48.

Ex vivo assays showed the metabolic activity in RPE to decrease with exposure to MSC‐CdM that was pretreated with antibodies to each trophic factor, except for CTGF (Fig. 6C). Similarly, RPE viability was reduced by antibody blockade of trophic factor receptors before CdM exposure (Fig. 6D). Total FITC‐labeled POS that bound (POS^Bound^) RPE increased during 24 hours of culture in GM compared with BM and was highest in MSC‐CdM (Fig. 6E–6H). Fluorescent images from POS^Bound^ (green), colocalized in silico with phagolysosome marker cathepsin‐D (red), demonstrated greater POS internalization in MSC‐CdM versus BM when normalized to cell number (DAPI, blue; Fig. 6J, 6K). bFGF is secreted by rat MSCs 49 and was reported sufficient to restore RPE phagocytic competence in RCS rat retinae 50. However, only modest diminution of POS^Bound^ was observed with bFGF neutralization of CdM (Fig. 6L). However, BDNF neutralization in CdM reduced POS^Bound^, which was restored with the addition of 50 ng/ml recombinant BDNF to BM (Fig. 6M).

### Blood Composition Changes Following MSC^IV^ Suggest Systemic Immunomodulation

**Figure 7:**
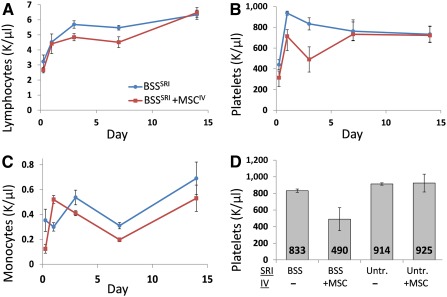
Systemic cellular changes after MSC^IV^. Postnatal day 28 Royal College of Surgeons rats received BSS^SRI^ supplemented with MSC^IV^ 24 hours later (*n* = 3) or no treatment (*n* = 3). Complete blood counts were performed on days 1, 3, 7, and 14. MSC^IV^‐treated rats showed reduced circulating lymphocytes on days 3 and 7 **(A)**, fewer platelets on day 3 **(B)**, and fewer monocytes on days 3 and 7 **(C)**. Two of the three rats that received MSC^IV^ displayed thrombocytopenia with platelet counts of 306 and 254 K/µl, well below the normal range (685–1,436 K/µl). Abbreviations: BSS, balanced salt solution; IV, intravenous; MSC, mesenchymal stem cell; SRI, subretinal injection; Untr., untreated.

In contrast to paracrine mechanisms of retinal protection from MSC^SRI^, we explored the possibility of systemic effects from MSC^IV^. We compared the blood cellular composition changes after MSC^IV^ using CBCs over 2 weeks. Rats received unilateral BSS^SRI^ alone or combined with MSC^IV^. Circulating platelets were reduced on day 3 (Fig. 7A), and lymphocytes were reduced on day 7 (Fig. 7B). Neutrophils were also decreased on day 3 from MSC^IV^‐supplemented BSS^SRI^ compared with BSS^SRI^ alone (197 ± 16 vs. 310 ± 25; *p* < .05; data not shown). Spleen weights normalized to body weight did not differ among the rats from the two treatment groups (data not shown). Two of the three rats that received MSC^IV^ had thrombocytopenia on day 3, with platelet counts of 306 × 10^9^/l and 254 × 10^9^/l, well below the normal range (685–1,436 × 10^9^/l). Monocyte counts were also reduced on days 3 and 7 after MSC^IV^ (Fig. 7C). CBCs from untreated animals and those that received MSC^IV^ alone and untreated with MSC^IV^ indicated that platelet reduction occurred exclusively when MSC^IV^ supplemented BSS^SRI^ (Fig. 7D).

## Discussion

Consistent with our previous findings, MSC^SRI^ alone mitigated retinal dystrophy in a focal manner proximal to the graft site and preserved corresponding vision in RCS rats. Sham treatment (BSS^SRI^) also produced minor focal rescue (Fig. 1F). The sham effect did not account for functional rescue from cell injections, because the efficacy from MSC^SRI^ was consistently greater than that from BSS^SRI^ (Fig. 1; supplemental online Fig. 1). In contrast, MSC^IV^ alone generated mild and widespread phenotypic protection. When combined, however, retinotopic mapping by LTR showed that MSC^IV^ supplementation extended the rescue area conferred by MSC^SRI^ and increased total retinal sensitivity (Fig. 1B; supplemental online Fig. 1). Synergistic vision rescue from combined MSC transplant suggested that MSC protection mechanisms are not redundant and differ by administration route. MSC^IV^ supplementation also augmented the rescue conferred by the sham effect (Fig. 1A, 1B). Frequently omitted from discussion, the sham effect became advantageous by its augmentation without retinal recruitment. This indicates that synergistic rescue is not limited to MSC^SRI^. The existence of the sham effect suggests that endogenous protection mechanisms might be augmented to reduce vision loss from disease or injury, which might, in fact, be represented by the increased visual acuity and luminance sensitivity after MSC^IV^ supplementation.

Vision rescue in the absence of MSC recruitment was demonstrated by CdM^SRI^ (Fig. 4A). Because the rescue (from CdM^SRI^) was greater than that from BSS^SRI^, the repertoire of MSC‐derived trophic factors likely differed from those induced by the sham effect or differed in quantity (Fig. 5). Vision rescue from MSC^SRI^ or BSS^SRI^ was similarly increased by MSC^IV^ supplementation (3.9‐fold with MSC^SRI^ and 3.4‐fold with BSS^SRI^). Rescue was greater from MSC^SRI^ by all functional tests, indicating that trophic support from BSS^SRI^ with MSC^IV^ did not compensate for the absence of MSC^SRI^ in retinae (Fig. 5F–5H). Because fewer MSC^IV^ were in retinae than from MSC^SRI^ (Fig. 5I), additional paracrine support from MSC^IV^ was unlikely to be the mechanism of synergistic rescue. This further supports distinct and cooperative protection mechanisms by MSCs and from the sham effect.

MSC‐secreted factors activate prolife signal transduction pathways in preapoptotic cells that are jeopardized by metabolic dysregulation from oxygen, glucose, or nutrient deprivation 51. Ex vivo assays showed that MSC‐CdM supports photoreceptor survival ex vivo 52 and enhances RPE internalization of POSs 53, with an increase in RPE metabolic viability (Fig. 6C, 6D). Rat MSCs have been shown to secrete BDNF 49, the transcriptional silencing of which 53, or antibody neutralization in MSC‐CdM (Fig. 6M), reduced RPE phagocytosis ability. Although not yet clear, a putative mechanism by which RPE phagocytosis might circumvent MERTK function was recently described to occur through opsonizing bridge molecules secreted by human umbilical tissue‐derived cells (hUTCs) 53. Transcript silencing of opsonizing peptides yielded hUTC‐CdM with reduced ability to promote POS uptake by cultured RPE cells derived from RCS rats. POS preincubation with recombinant opsonizing peptides increased phagocytosis, as did recombinant growth factor addition. It will be interesting to determine which alternate membrane receptors can be used by opsonizing peptides, and whether they compensate for MERTK dysfunction in vivo.

A role for NGF signaling was recently implicated for endogenous MSC activation of Müller glia 54 in which CNTF expression was shown to be instigated by exogenous MSC^IV^ 20. CNTF secretion by encapsulated RPE was clinically shown to preserve vision 55. Furthermore, photoreceptor survival signaling has been proposed to occur through intermediary cells that regulate retinal homeostasis. Müller glia cells fit this description as they have been described to increase NGF, CNTF, and GDNF expression in response to infiltrative microglia following injury, the receptors for which are absent on photoreceptors 56. These data are consistent with the role for activated Müller glia as a conduit for trophic support of photoreceptor survival and, collectively, support a cell signaling network mechanism for MSC‐mediated retinal protection.

Because the MSC secretome is environment‐dependent 57, 58, 59, 60, 61, the administration route likely influenced the different MSC responses 62, which might partially account for the nonredundant mechanisms. Intravenous delivery provided initial access to circulating immune cells. MSC^IV^‐secreted peptides have been shown to decrease circulating inflammatory cell numbers 63 and inflammatory responses from circulating lymphocytes 64, macrophages 65, and dendritic cells 66. MSC influence on regulatory T cells was suggested as the mechanism by which MSCs promote allograft tolerance 67 and ameliorate autoimmune uveoretinitis 68. Immune cell regulation is consistent with the role of MSCs and their progeny, which comprise stromal elements of endosteal and sinusoidal niches that support hematopoiesis in vivo 69 and ectopic HSC niche formation de novo 70, 71. The orthotopic locale of MSCs is the marrow space, in which PKH26^+^ MSCs were detected on day 3 after MSC^IV^, albeit infrequently (supplemental online Fig. 2B).

CXCL12/CXCR4 signaling in HSC niches also influences lymphoid and myeloid cell development and mobilization and retention in bone marrow 72 and might explain our observed lymphocyte and/or platelet decrease after MSC^IV^ (Fig. 7). For example, MSC‐derived osteoblasts were shown to inhibit megakaryocyte maturation in vivo 73 and to influence platelet biogenesis ex vivo 74, a process that coincides with the timeframe of our observed platelet reduction after MSC^IV^. The platelet decrease occurred only in the context of BSS^SRI^, which might suggest an injury‐specific systemic response that does not require retinal recruitment (Fig. 7D). Blood composition changes were previously reported at 6 and 24 hours after MSC^IV^ supplementation of cerebral infarction. Only brief MSC persistence was noted, but prolonged functional efficacy coincided with sustained activation of microglia and astrocytes proximal to the injury 75.

MSCs were detected in the circulation 3 days after MSC^IV^ but were absent from retinal cryosections (supplemental online Figure 2C; Fig. 5F). MSCs were sporadically detected in retinae by day 7 (Fig. 5H). Latent chemotaxis, however, was unlikely, because MSCs were not detected in P60 or P90 retinae (data not shown). This instead suggests passive MSC migration or migration to alternative sites. Furthermore, CXCL12 conditioning did not increase MSC^IV^ recruitment at 24 or 72 hours, the typical timeframe for MSC chemotaxis, despite improved ERG and OKR (Fig. 4). Similar proliferation and differentiation after CXCL12 conditioning excluded a cell dose (Fig. 2L) and multipotency (Fig. 3) advantage to which improvement could be attributed.

It is possible that MSC^IV^‐secreted factors acted on retinae by endocrine means 76 (i.e., passive MSC^IV^ aggregation at thrombotic capillary beds from which trophic factors are secreted into the circulation). We did not examine this possibility; however, in such a scenario, intra‐arterial infusion might increase the number of aggregated MSCs and improve rescue. The intent of enhancing MSC^IV^ migration, targeting, adherence, or integration to specific tissues is to improve therapeutic efficacy 62. However, the sparsity of homed MSC^IV^ in the presence of vision rescue suggests that the site of injury is not the only site of MSC action. Accordingly, enhanced MSC^IV^ migration to the retina might reduce vision rescue if it is at the expense of recruitment to the appropriate destination from which MSCs confer therapeutic efficacy. Additionally, the duration of vision rescue greatly exceeded that of MSC^SRI^ graft survival. It is therefore of interest to characterize the temporal window during which synergistic rescue can be achieved and whether this timeframe can be extended by altering the transplant strategy (i.e., MSC^IV^ redosing).

MSC^IV^ supplementation did not facilitate MSC^SRI^ persistence in the subretinal space. This was unlikely to be due to rejection, because MSCs are hypoimmunogenic, mitigate inflammatory responses 62, and support the RCS‐RPE regulation of subretinal immune privilege 77. Rejection‐independent mechanisms are further supported by the immune tolerance of isogenic 19 and syngeneic retinal grafts without immune suppression 78 and by MSC graft dissolution in immunodeficient animals 79. Furthermore, we observed long‐term retinal persistence of human RPE 33, NPCs 31, and iNPCs in RCS retinae 32. Donor cell persistence was unlikely from dysfunctional *MerTK* in macrophages, because engulfment can occur through alternate pathways 80, 81. In contrast, MSC^IV^ has been shown to reduce microglia/macrophage infiltration to ischemic sites 82 and to polarize macrophages toward a less inflammatory state 83, 84, 85. Collectively, these observations suggest that MSC^SRI^ disappearance from dystrophic retinae is specific to MSCs. Yet unclear is whether this can be advantageous for cotransplant paradigms that necessitate transient support.

Using MSC^IV^ as adjuvant therapy is particularly attractive because MSCs are a renewable resource that can be readministered noninvasively to minimize retinal damage in patients. MSC^IV^ could also reduce immunosuppression toxicity by permitting lower dosing regimens and help avoid complications from patient noncompliance. The multiple and complex mechanisms described for MSC‐mediated retinal protection require further clarification. It is not yet clear how MSC coadministration synergizes to protect dystrophic retinae. However, this observation underscores the possibility that yet unidentified transplant strategies might improve MSC therapy. Of clinical relevance is whether MSC^IV^ supplementation can improve vision rescue from different disease etiologies. More generally, MSC^IV^ adjuvant therapy may enhance the therapeutic outcomes of cell‐based therapies for other medical conditions.

## Conclusion

MSC^IV^ has gained momentum for clinical adoption with their immunosuppressive properties serving as the preeminent rationale. The vision rescue from MSC^SRI^ in RCS rats was dramatically increased by MSC^IV^ supplementation. Thus, cell therapies for progressive degenerative diseases, such as AMD and RP, might be more effective when combined with MSC^IV^. The translational implications of potentiating treatments currently deemed insufficient warrants further investigation of MSC^IV^ adjuvant therapy.

## Author Contributions

B.B. and S.W.: conception and design, collection and/or assembly of data, data analysis and interpretation, manuscript writing, final approval of manuscript, financial support; S.G.: collection and/or assembly of data, data analysis and interpretation; B.L.: conception and design, collection and/or assembly of data, data analysis and interpretation.

## Disclosure of Potential Conflicts of Interest

The authors indicated no potential conflicts of interest.

## Supporting information

Supporting Information

## References

[1] Lim LS , Mitchell P , Seddon JM et al. Age‐related macular degeneration. Lancet 2012;379:1728–1738. 22559899 10.1016/S0140-6736(12)60282-7

[2] Hartong DT , Berson EL , Dryja TP . Retinitis pigmentosa. Lancet 2006;368:1795–1809. 17113430 10.1016/S0140-6736(06)69740-7

[3] Consumer Information on Stem Cells. January 6, 2012. Available at http://www.fda.gov/newsevents/publichealthfocus/ucm286218.htm. Accessed December 15, 2015

[4] Little MT , Storb R . History of haematopoietic stem‐cell transplantation. Nat Rev Cancer 2002;2:231–238. 11990860 10.1038/nrc748

[5] Osiris Therapeutics I. World's First Approved Stem Cell Drug—Osiris Receives Marketing Clearance from Health Canada for Prochymal. May 17, 2012. Available at http://files.shareholder.com/downloads/OSIR/1250455046x0x570475/DDD9C94F‐D23A‐4986‐9EE1‐9FE626BDF07E/OSIR_News_2012_5_17_General.pdf. Accessed February 15, 2016

[6] Syed BA , Evans JB . Stem cell therapy market. Nat Rev Drug Discov 2013;12:185–186. 23449299 10.1038/nrd3953

[7] Huang H , He J , Teng X et al. Combined intrathymic and intravenous injection of mesenchymal stem cells can prolong the survival of rat cardiac allograft associated with decrease in miR‐155 expression. J Surg Res 2013;185:896–903. 23870834 10.1016/j.jss.2013.06.015

[8] Oh JY , Lee RH , Yu JM et al. Intravenous mesenchymal stem cells prevented rejection of allogeneic corneal transplants by aborting the early inflammatory response. Mol Ther 2012;20:2143–2152. 22929658 10.1038/mt.2012.165 PMC3498800

[9] Bartholomew A , Sturgeon C , Siatskas M et al. Mesenchymal stem cells suppress lymphocyte proliferation in vitro and prolong skin graft survival in vivo. Exp Hematol 2002;30:42–48. 11823036 10.1016/s0301-472x(01)00769-x

[10] Beutelspacher SC , Pillai R , Watson MP et al. Function of indoleamine 2,3‐dioxygenase in corneal allograft rejection and prolongation of allograft survival by over‐expression. Eur J Immunol 2006;36:690–700. 16482510 10.1002/eji.200535238

[11] Noort WA , Kruisselbrink AB , in't Anker PS et al. Mesenchymal stem cells promote engraftment of human umbilical cord blood‐derived CD34(+) cells in NOD/SCID mice. Exp Hematol 2002;30:870–878. 12160838 10.1016/s0301-472x(02)00820-2

[12] Gal A , Li Y , Thompson DA et al. Mutations in MERTK, the human orthologue of the RCS rat retinal dystrophy gene, cause retinitis pigmentosa. Nat Genet 2000;26:270–271. 11062461 10.1038/81555

[13] D'Cruz PM , Yasumura D , Weir J et al. Mutation of the receptor tyrosine kinase gene Mertk in the retinal dystrophic RCS rat. Hum Mol Genet 2000;9:645–651. 10699188 10.1093/hmg/9.4.645

[14] Dowling JE , Sidman RL . Inherited retinal dystrophy in the rat. J Cell Biol 1962;14:73–109. 13887627 10.1083/jcb.14.1.73 PMC2106090

[15] LaVail MM . Legacy of the RCS rat: Impact of a seminal study on retinal cell biology and retinal degenerative diseases. Prog Brain Res 2001;131:617–627. 11420975 10.1016/s0079-6123(01)31048-8

[16] Leow SN , Luu CD , Hairul Nizam MH et al. Safety and efficacy of human Wharton's jelly‐derived mesenchymal stem cells therapy for retinal degeneration. PLoS One 2015;10:e0128973. 26107378 10.1371/journal.pone.0128973 PMC4479609

[17] Lund RD , Wang S , Lu B et al. Cells isolated from umbilical cord tissue rescue photoreceptors and visual functions in a rodent model of retinal disease. Stem Cells 2007;25:602–611. 17053209 10.1634/stemcells.2006-0308

[18] Lu B , Wang S , Girman S et al. Human adult bone marrow‐derived somatic cells rescue vision in a rodent model of retinal degeneration. Exp Eye Res 2010;91:449–455. 20603115 10.1016/j.exer.2010.06.024

[19] McGill TJ , Lund RD , Douglas RM et al. Syngeneic Schwann cell transplantation preserves vision in RCS rat without immunosuppression. Invest Ophthalmol Vis Sci 2007;48:1906–1912. 17389527 10.1167/iovs.06-1117

[20] Wang S , Lu B , Girman S et al. Non‐invasive stem cell therapy in a rat model for retinal degeneration and vascular pathology. PLoS One 2010;5:e9200. 20169166 10.1371/journal.pone.0009200 PMC2821411

[21] Wen R , Song Y , Cheng MT et al. Injury‐induced upregulation of bFGF and CNTF mRNAS in the rat retina. J Neurosci 1995;11:7377–7385. 10.1523/JNEUROSCI.15-11-07377.1995 PMC6578062 7472491

[22] Cao W , Li F , Steinberg RH et al. Development of normal and injury‐induced gene expression of aFGF, bFGF, CNTF, BDNF, GFAP and IGF‐I in the rat retina. Exp Eye Res 2001;72:591–604. 11311051 10.1006/exer.2001.0990

[23] Li Y , Atmaca‐Sonmez P , Schanie CL et al. Endogenous bone marrow derived cells express retinal pigment epithelium cell markers and migrate to focal areas of RPE damage. Invest Ophthalmol Vis Sci 2007;48:4321–4327. 17724223 10.1167/iovs.06-1015

[24] Machalińska A , Kłos P , Baumert B et al. Stem cells are mobilized from the bone marrow into the peripheral circulation in response to retinal pigment epithelium damage—A pathophysiological attempt to induce endogenous regeneration. Curr Eye Res 2011;36:663–672. 21657828 10.3109/02713683.2011.576796

[25] Goldenberg‐Cohen N , Avraham‐Lubin BC , Sadikov T et al. Effect of coadministration of neuronal growth factors on neuroglial differentiation of bone marrow‐derived stem cells in the ischemic retina. Invest Ophthalmol Vis Sci 2014;55:502–512. 24370836 10.1167/iovs.13-12223

[26] Xu W , Wang XT , Xu GX et al. Stromal cell‐derived factor 1α‐stimulated mesenchymal stem cells confer enhanced protection against light‐induced retinal degeneration in rats. Curr Eye Res 2014;39:69–78. 24074164 10.3109/02713683.2013.824988

[27] Gao J , Dennis JE , Muzic RF et al. The dynamic in vivo distribution of bone marrow‐derived mesenchymal stem cells after infusion. Cells Tissues Organs 2001;169:12–20. 11340257 10.1159/000047856

[28] Castanheira P , Torquetti L , Nehemy MB et al. Retinal incorporation and differentiation of mesenchymal stem cells intravitreally injected in the injured retina of rats. Arq Bras Oftalmol 2008;71:644–650. 19039457 10.1590/s0004-27492008000500007

[29] Kicic A , Shen WY , Wilson AS et al. Differentiation of marrow stromal cells into photoreceptors in the rat eye. J Neurosci 2003;23:7742–7749. 12944502 10.1523/JNEUROSCI.23-21-07742.2003 PMC6740611

[30] Arnhold S , Heiduschka P , Klein H et al. Adenovirally transduced bone marrow stromal cells differentiate into pigment epithelial cells and induce rescue effects in RCS rats. Invest Ophthalmol Vis Sci 2006;47:4121–4129. 16936132 10.1167/iovs.04-1501

[31] Wang S , Girman S , Lu B et al. Long‐term vision rescue by human neural progenitors in a rat model of photoreceptor degeneration. Invest Ophthalmol Vis Sci 2008;49:3201–3206. 18579765 10.1167/iovs.08-1831 PMC3055787

[32] Tsai Y , Lu B , Bakondi B et al. Human iPSC‐derived neural progenitors preserve vision in an AMD‐like model. Stem Cells 2015;33:2537–2549. 25869002 10.1002/stem.2032 PMC5477659

[33] Lu B , Malcuit C , Wang S et al. Long‐term safety and function of RPE from human embryonic stem cells in preclinical models of macular degeneration. Stem Cells 2009;27:2126–2135. 19521979 10.1002/stem.149

[34] Bakondi B , Shimada IS , Perry A et al. CD133 identifies a human bone marrow stem/progenitor cell sub‐population with a repertoire of secreted factors that protect against stroke. Mol Ther 2009;17:1938–1947. 19690521 10.1038/mt.2009.185 PMC2835040

[35] Girman SV , Wang S , Lund RD . Time course of deterioration of rod and cone function in RCS rat and the effects of subretinal cell grafting: A light‐ and dark‐adaptation study. Vision Res 2005;45:343–354. 15607350 10.1016/j.visres.2004.08.023

[36] Wynn RF , Hart CA , Corradi‐Perini C et al. A small proportion of mesenchymal stem cells strongly expresses functionally active CXCR4 receptor capable of promoting migration to bone marrow. Blood 2004;104:2643–2645. 15251986 10.1182/blood-2004-02-0526

[37] Shi M , Li J , Liao L et al. Regulation of CXCR4 expression in human mesenchymal stem cells by cytokine treatment: Role in homing efficiency in NOD/SCID mice. Haematologica 2007;92:897–904. 17606439 10.3324/haematol.10669

[38] Hung SC , Pochampally RR , Hsu SC et al. Short‐term exposure of multipotent stromal cells to low oxygen increases their expression of CX3CR1 and CXCR4 and their engraftment in vivo. PLoS One 2007;2:e416. 17476338 10.1371/journal.pone.0000416 PMC1855077

[39] Iso Y , Spees JL , Serrano C et al. Multipotent human stromal cells improve cardiac function after myocardial infarction in mice without long‐term engraftment. Biochem Biophys Res Commun 2007;354:700–706. 17257581 10.1016/j.bbrc.2007.01.045 PMC1851899

[40] Kinnaird T , Stabile E , Burnett MS et al. Marrow‐derived stromal cells express genes encoding a broad spectrum of arteriogenic cytokines and promote in vitro and in vivo arteriogenesis through paracrine mechanisms. Circ Res 2004;94:678–685. 14739163 10.1161/01.RES.0000118601.37875.AC

[41] Gnecchi M , He H , Liang OD et al. Paracrine action accounts for marked protection of ischemic heart by Akt‐modified mesenchymal stem cells. Nat Med 2005;11:367–368. 15812508 10.1038/nm0405-367

[42] Faktorovich EG , Steinberg RH , Yasumura D et al. Photoreceptor degeneration in inherited retinal dystrophy delayed by basic fibroblast growth factor. Nature 1990;347:83–86. 2168521 10.1038/347083a0

[43] Touchard E , Heiduschka P , Berdugo M et al. Non‐viral gene therapy for GDNF production in RCS rat: The crucial role of the plasmid dose. Gene Ther 2012;19:886–898. 21993171 10.1038/gt.2011.154

[44] Zhang M , Mo X , Fang Y et al. Rescue of photoreceptors by BDNF gene transfer using in vivo electroporation in the RCS rat of retinitis pigmentosa. Curr Eye Res 2009;34:791–799. 19839873 10.1080/02713680903086018

[45] Lenzi L , Coassin M , Lambiase A et al. Effect of exogenous administration of nerve growth factor in the retina of rats with inherited retinitis pigmentosa. Vision Res 2005;45:1491–1500. 15781068 10.1016/j.visres.2004.12.020

[46] Otsuka H , Arimura N , Sonoda S et al. Stromal cell‐derived factor‐1 is essential for photoreceptor cell protection in retinal detachment. Am J Pathol 2010;177:2268–2277. 20889568 10.2353/ajpath.2010.100134 PMC2966786

[47] Chen CL , Liang CM , Chen YH et al. Bevacizumab modulates epithelial‐to‐mesenchymal transition in the retinal pigment epithelial cells via connective tissue growth factor up‐regulation. Acta Ophthalmol 2012;90:e389–e398. 22712616 10.1111/j.1755-3768.2012.02426.x

[48] Iso Y , Rao KS , Poole CN et al. Priming with ligands secreted by human stromal progenitor cells promotes grafts of cardiac stem/progenitor cells after myocardial infarction. Stem Cells 2014;32:674–683. 24022988 10.1002/stem.1546 PMC3966427

[49] Kendirci M , Trost L , Bakondi B et al. Transplantation of nonhematopoietic adult bone marrow stem/progenitor cells isolated by p75 nerve growth factor receptor into the penis rescues erectile function in a rat model of cavernous nerve injury. J Urol 2010;184:1560–1566. 20728109 10.1016/j.juro.2010.05.088 PMC3014289

[50] McLaren MJ , Inana G . Inherited retinal degeneration: Basic FGF induces phagocytic competence in cultured RPE cells from RCS rats. FEBS Lett 1997;412:21–29. 9257682 10.1016/s0014-5793(97)00566-8

[51] Murphy MB , Moncivais K , Caplan AI . Mesenchymal stem cells: Environmentally responsive therapeutics for regenerative medicine. Exp Mol Med 2013;45:e54. 24232253 10.1038/emm.2013.94 PMC3849579

[52] Inoue Y , Iriyama A , Ueno S et al. Subretinal transplantation of bone marrow mesenchymal stem cells delays retinal degeneration in the RCS rat model of retinal degeneration. Exp Eye Res 2007;85:234–241. 17570362 10.1016/j.exer.2007.04.007

[53] Cao J , Murat C , An W et al. Human umbilical tissue‐derived cells rescue retinal pigment epithelium dysfunction in retinal degeneration. Stem Cells 2016;34:367–379. 26523756 10.1002/stem.2239

[54] Jian Q , Li Y , Yin ZQ . Rat BMSCs initiate retinal endogenous repair through NGF/TrkA signaling. Exp Eye Res 2015;132:34–47. 25584870 10.1016/j.exer.2015.01.008

[55] Zhang K , Hopkins JJ , Heier JS et al. Ciliary neurotrophic factor delivered by encapsulated cell intraocular implants for treatment of geographic atrophy in age‐related macular degeneration. Proc Natl Acad Sci USA 2011;108:6241–6245. 21444807 10.1073/pnas.1018987108 PMC3076847

[56] Harada T , Harada C , Mitamura Y et al. Neurotrophic factor receptors in epiretinal membranes after human diabetic retinopathy. Diabetes Care 2002;25:1060–1065. 12032115 10.2337/diacare.25.6.1060

[57] Liu Y , Dulchavsky DS , Gao X et al. Wound repair by bone marrow stromal cells through growth factor production. J Surg Res 2006;136:336–341. 17045612 10.1016/j.jss.2006.07.037

[58] Johnson TV , DeKorver NW , Levasseur VA et al. Identification of retinal ganglion cell neuroprotection conferred by platelet‐derived growth factor through analysis of the mesenchymal stem cell secretome. Brain 2014;137:503–519. 24176979 10.1093/brain/awt292 PMC3914467

[59] Potier E , Ferreira E , Andriamanalijaona R et al. Hypoxia affects mesenchymal stromal cell osteogenic differentiation and angiogenic factor expression. Bone 2007;40:1078–1087. 17276151 10.1016/j.bone.2006.11.024

[60] Chen X , Katakowski M , Li Y et al. Human bone marrow stromal cell cultures conditioned by traumatic brain tissue extracts: Growth factor production. J Neurosci Res 2002;69:687–691. 12210835 10.1002/jnr.10334

[61] Ohnishi S , Yasuda T , Kitamura S et al. Effect of hypoxia on gene expression of bone marrow‐derived mesenchymal stem cells and mononuclear cells. Stem Cells 2007;25:1166–1177. 17289933 10.1634/stemcells.2006-0347

[62] Kean TJ , Lin P , Caplan AI et al. MSCs: Delivery routes and engraftment, cell‐targeting strategies, and immune modulation. Stem Cells Int 2013;2013:732742. 24000286 10.1155/2013/732742 PMC3755386

[63] Yagi H , Soto‐Gutierrez A , Navarro‐Alvarez N et al. Reactive bone marrow stromal cells attenuate systemic inflammation via sTNFR1. Mol Ther 2010;18:1857–1864. 20664529 10.1038/mt.2010.155 PMC2951565

[64] Koning JJ , Kooij G , de Vries HE et al. Mesenchymal stem cells are mobilized from the bone marrow during inflammation. Front Immunol 2013;4:49. 23459632 10.3389/fimmu.2013.00049 PMC3586765

[65] Németh K , Leelahavanichkul A , Yuen PS et al. Bone marrow stromal cells attenuate sepsis via prostaglandin E(2)‐dependent reprogramming of host macrophages to increase their interleukin‐10 production. Nat Med 2009;15:42–49. 19098906 10.1038/nm.1905 PMC2706487

[66] Popp FC , Eggenhofer E , Renner P et al. Mesenchymal stem cells can induce long‐term acceptance of solid organ allografts in synergy with low‐dose mycophenolate. Transpl Immunol 2008;20:55–60. 18762258 10.1016/j.trim.2008.08.004

[67] Obermajer N , Popp FC , Soeder Y et al. Conversion of Th17 into IL‐17A(neg) regulatory T cells: A novel mechanism in prolonged allograft survival promoted by mesenchymal stem cell‐supported minimized immunosuppressive therapy. J Immunol 2014;193:4988–4999. 25305313 10.4049/jimmunol.1401776

[68] Li G , Yuan L , Ren X et al. The effect of mesenchymal stem cells on dynamic changes of T cell subsets in experimental autoimmune uveoretinitis. Clin Exp Immunol 2013;173:28–37. 23607419 10.1111/cei.12080 PMC3694532

[69] Wilson A , Trumpp A . Bone‐marrow haematopoietic‐stem‐cell niches. Nat Rev Immunol 2006;6:93–106. 16491134 10.1038/nri1779

[70] Bakondi B , Spees JL . Human CD133‐derived bone marrow stromal cells establish ectopic hematopoietic microenvironments in immunodeficient mice. Biochem Biophys Res Commun 2010;400:212–218. 20719235 10.1016/j.bbrc.2010.08.040 PMC2942979

[71] Sacchetti B , Funari A , Michienzi S et al. Self‐renewing osteoprogenitors in bone marrow sinusoids can organize a hematopoietic microenvironment. Cell 2007;131:324–336. 17956733 10.1016/j.cell.2007.08.025

[72] Kucia M , Jankowski K , Reca R et al. CXCR4‐SDF‐1 signalling, locomotion, chemotaxis and adhesion. J Mol Histol 2004;35:233–245. 15339043 10.1023/b:hijo.0000032355.66152.b8

[73] Pallotta I , Lovett M , Rice W et al. Bone marrow osteoblastic niche: A new model to study physiological regulation of megakaryopoiesis. PLoS One 2009;4:e8359. 20027303 10.1371/journal.pone.0008359 PMC2793008

[74] Liu M , Yang SG , Shi L et al. Mesenchymal stem cells from bone marrow show a stronger stimulating effect on megakaryocyte progenitor expansion than those from non‐hematopoietic tissues. Platelets 2010;21:199–210. 20187717 10.3109/09537101003602483

[75] Acosta SA , Tajiri N , Shinozuka K et al. Long‐term upregulation of inflammation and suppression of cell proliferation in the brain of adult rats exposed to traumatic brain injury using the controlled cortical impact model. PLoS One 2013;8:e53376. 23301065 10.1371/journal.pone.0053376 PMC3536766

[76] Lee RH , Pulin AA , Seo MJ et al. Intravenous hMSCs improve myocardial infarction in mice because cells embolized in lung are activated to secrete the anti‐inflammatory protein TSG‐6. Cell Stem Cell 2009;5:54–63. 19570514 10.1016/j.stem.2009.05.003 PMC4154377

[77] Xian B , Huang B . The immune response of stem cells in subretinal transplantation. Stem Cell Res Ther 2015;6:161. 26364954 10.1186/s13287-015-0167-1 PMC4568575

[78] Anosova NG , Illigens B , Boisgérault F et al. Antigenicity and immunogenicity of allogeneic retinal transplants. J Clin Invest 2001;108:1175–1183. 11602625 10.1172/JCI12204 PMC209524

[79] Rosová I , Dao M , Capoccia B et al. Hypoxic preconditioning results in increased motility and improved therapeutic potential of human mesenchymal stem cells. Stem Cells 2008;26:2173–2182. 18511601 10.1634/stemcells.2007-1104 PMC3017477

[80] Gery I , O'Brien PJ . RCS rat macrophages exhibit normal ROS phagocytosis. Invest Ophthalmol Vis Sci 1981;20:675–679. 7216681

[81] Seitz HM , Camenisch TD , Lemke G et al. Macrophages and dendritic cells use different Axl/Mertk/Tyro3 receptors in clearance of apoptotic cells. J Immunol 2007;178:5635–5642. 17442946 10.4049/jimmunol.178.9.5635

[82] Li Y , Chen J , Zhang CL et al. Gliosis and brain remodeling after treatment of stroke in rats with marrow stromal cells. Glia 2005;49:407–417. 15540231 10.1002/glia.20126

[83] Abumaree MH , Al Jumah MA , Kalionis B et al. Human placental mesenchymal stem cells (pMSCs) play a role as immune suppressive cells by shifting macrophage differentiation from inflammatory M1 to anti‐inflammatory M2 macrophages. Stem Cell Rev 2013;9:620–641. 23812784 10.1007/s12015-013-9455-2

[84] Cho DI , Kim MR , Jeong HY et al. Mesenchymal stem cells reciprocally regulate the M1/M2 balance in mouse bone marrow‐derived macrophages. Exp Mol Med 2014;46:e70. 24406319 10.1038/emm.2013.135 PMC3909888

[85] Ti D , Hao H , Tong C et al. LPS‐preconditioned mesenchymal stromal cells modify macrophage polarization for resolution of chronic inflammation via exosome‐shuttled let‐7b. J Transl Med 2015;13:308. 26386558 10.1186/s12967-015-0642-6 PMC4575470

