# Supplementary material for: Multimodal Delivery of Isogenic Mesenchymal Stem Cells Yields Synergistic Protection from Retinal Degeneration and Vision Loss
Source: Stem Cells Transl Med. 2016 Sep 9;6(2):444–57. doi: 10.5966/sctm.2016-0181 (PMC5442813; doi:10.5966/sctm.2016-0181)
Supplement: Supplementary file 1 — Supporting Information [file SCT3-6-444-s001.pdf]

Supplemental Figures – Wang et al.

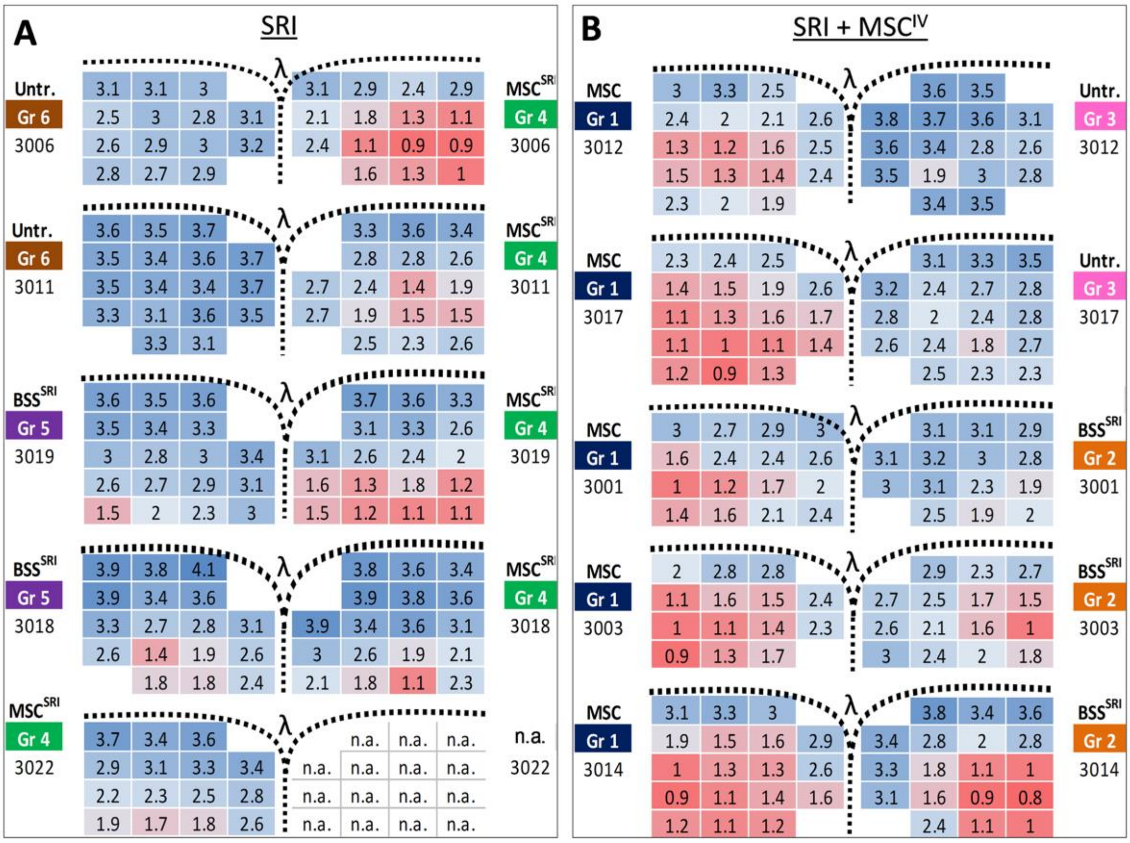

Figure S1. Retinotopic Sensitivity Mapping from LTR in Individual Animals.

LTR in P90-95 rats that received subretinal treatment only (**A**), or in combination with MSC^{IV} (**B**) show detailed retinotopic maps of retinal sensitivity (corresponding with **Fig. 1B)**. Hot spots of retinal sensitivity to light stimulation are shown by heat map overlay in red, defined as values below 33% of the mean over all experimental treatments.

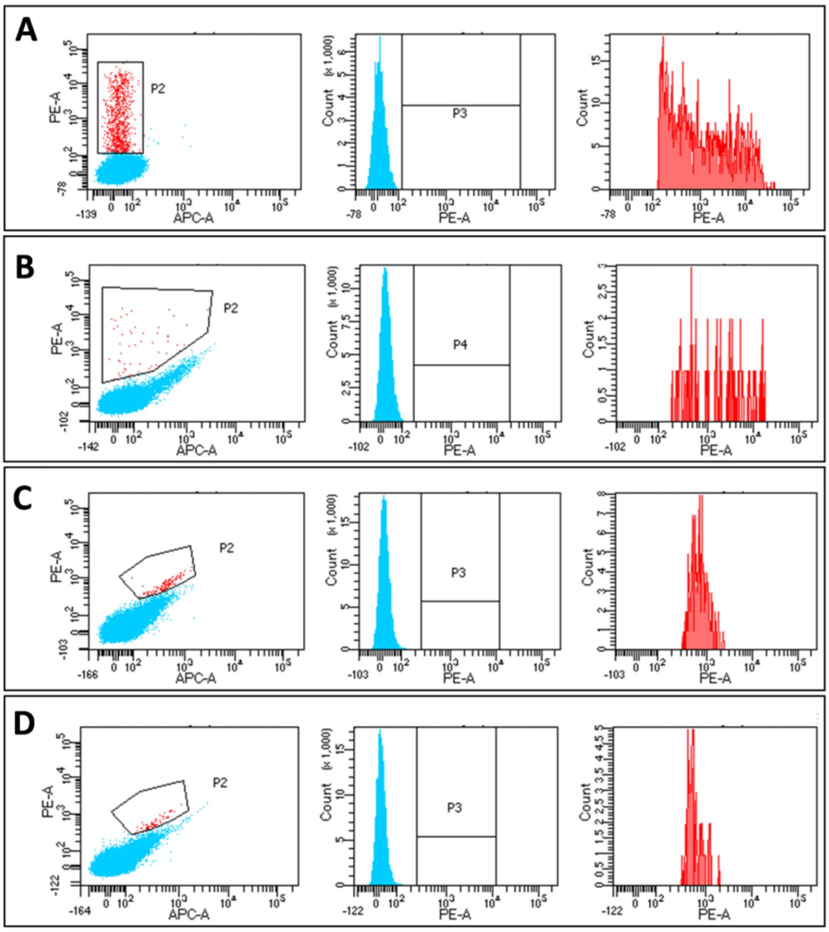

Figure S2. MSC^{IV} Detection in Tissues. Representative flow cytometry data showing PKH26^{+} MSC detection (red) 3 days post-MSC^{IV} in peripheral blood (**A**), bone marrow aspirate from femur (**B**), and enzymatically dissociated BSS^{SRI}-treated eye (**C**) or untreated contralateral eye **(D**). Representative number of MSCs detected in retinae after debris, doublet, and autofluorescent cell exclusion was 164 (of parent 276,635; 0.06%, **C**) and 62 (of parent 231,049; 0.03%, **D**).
